# Seeding the Infant Gut in Early Life—Effects of Maternal and Infant Seeding with Probiotics on Strain Transfer, Microbiota, and Gastrointestinal Symptoms in Healthy Breastfed Infants

**DOI:** 10.3390/nu15184000

**Published:** 2023-09-15

**Authors:** Cathrine Melsaether, Diana Høtoft, Anja Wellejus, Gerben D. A. Hermes, Anders Damholt

**Affiliations:** 1 Chr. Hansen A/S, Boege Alle 10-12, 2970 Hoersholm, Denmark; dkawe@chr-hansen.com (A.W.); dkgerh@chr-hansen.com (G.D.A.H.); dkanda@chr-hansen.com (A.D.); 2 Department of Gynecology and Obstetrics, Aarhus University Hospital, Palle Juul-Jensens Boulevard 99, 8200 Aarhus, Denmark; diahoe@rm.dk

**Keywords:** *Bifidobacterium longum* subsp. *infantis* Bifin02 (DSM33361) (ISTILOS™), *Bifidobacterium animalis* subsp. *lactis* BB-12 (DSM15954) (BB-12^®^), *Lacticaseibacillus rhamnosus* LGG (DSM33156) (LGG^®^), *Lactobacillus acidophilus* LA-5 (DSM13241) (LA-5^®^), infants, probiotics, gut microbiome, gastrointestinal symptoms

## Abstract

We investigated the effects of two dosing regimens of two multi-strain probiotic products on the gut microbiota of breastfed infants, including the transfer of the dosed strains and clinical outcomes. In forty-seven dyads, infants were either exposed through maternal intake (MS) of *Lactobacillus acidophilus* LA-5, *Bifidobacterium animalis* subsp. *lactis* BB-12, *Lacticaseibacillus rhamnosus* LGG, and *Bifidobacterium longum* subsp. *infantis* Bifin02 from gestational week thirty-three until four weeks after birth (*n* = 24) or dosed directly (IS) with the same strains except for LA-5 starting within 24 h after birth until day 28 (*n* = 23). Infant stool samples were collected on day 0, 14, 28, and 42 after birth. Gastrointestinal symptoms were assessed by parents using an electronic diary. Microbiota composition was determined using 16S rRNA sequencing, and strain recovery was analyzed by qPCR. Notably, 100% of the IS infants were colonized with Bifin02 after 14 days as opposed to only 25% of the MS infants. Mean stool frequency was significantly lower in IS infants compared to MS infants and IS infants had softer stools on day 14, 28, and 42. A significantly steeper slope of progression of inconsolable crying and fussing was observed in MS infants compared to IS infants. In conclusion, direct infant seeding induced a faster increase in fecal bifidobacteria abundancy and Bifin02 recovery compared to dosed through the maternal intake.

## 1. Introduction

Emerging knowledge has consolidated the importance of a *Bifidobacterium*-dominated microbiome in early infancy and the associated potential health benefits in later life, such as a reduced risk of developing allergies [1,2] and other immune-mediated diseases, such as Type-1 diabetes and Crohn’s disease [3]. 

Bifidobacterial dominance in the infant gut, including the ability of the bifidobacteria to colonize during the neonatal period, is affected by the mode of delivery, e.g., vaginal birth or caesarean section [4], antibiotic use [5,6] and feeding mode [7,8]. Breastfeeding provides the infant with a unique composition of essential nutrients and is considered the optimal feeding mode for infants. In addition, breastmilk also contains undigestible human milk oligosaccharides (HMOs), which are polymers of simple sugars that serve as substrates for specific bacterial species and thereby play a key role in shaping the infant gut microbiota. In vaginally born, breast-fed infants that did not receive antibiotics, four species typically dominated the infant microbiota: *Bifidobacterium longum* subsp. *infantis* (*B. infantis*), *Bifidobacterium bifidum*, *Bifidobacterium breve*, and *Bifidobacterium longum* subsp. *longum* [9,10,11]. Strains of these species are all equipped with species-specific gene sets for HMO utilization, although intra-species variations are observed [12,13]. *B. infantis* has been acknowledged to hold one of the largest HMO clusters in its genome, which provides it with a competitive potential that increases colonization success in the infant gut [14]. During HMO utilization by bifidobacteria, metabolites involved in healthy infant immune and gut maturation have been identified, such as short chain fatty acids [15], folate [16], and aromatic lactic acids [17]. This emphasizes the importance of breastfeeding as the ideal food for infants and an effective way to ensure child health and survival. In addition, several papers have shown beneficial effects on infant health by supplementing with *B. infantis*, specifically in the context of managing underweight infants and immune development [17,18,19]. Interestingly, in one study, a native identified strain was genetically better adapted to grow on a broader set of saccharides, providing superior fitness over the dosed strain [18]. Besides the impact on the maturation of the gastrointestinal tract and the link to immune maturation, supplementation with bifidobacteria is associated with decreased crying and fussing in infants diagnosed with infantile colic [20,21,22]. In a recent systematic review and a Cochrane review on the topic, the use of probiotics for treatment of colic seems to be substantiated, whereas no clear evidence with regards to prevention of colic exists [23,24]. The definition for clinical diagnosis of infantile colic includes recurrent and prolonged periods of inconsolable crying, fussing, and irritability without any obvious cause or underlying disease. These criteria were revised by the Rome foundation and no longer specify the minimum crying and fussing time [25]. Moreover, infant colic has been associated with dysbiosis caused by an increased abundance of proteobacteria and a decreased abundance of bifidobacteria [22,26,27]. These data support the hypothesis that infantile colic may be caused by alterations in the infant gut microbiota which may lead to abnormal gut motility and gas production [28]. Inconsolable crying bouts and gastrointestinal discomfort are included as some of the main reasons for parents’ and caregivers’ concerns and may have a major impact on the establishment and quality of life of a new family. Moreover, these symptoms are associated with maternal post-partum depression, parental sense of guilt and frustration, early cessation of breastfeeding, and a more frequent number of health care provider visits [29,30]. 

The first major bacterial encounter of an infant is of maternal origin and occurs during and directly after vaginal birth [31]. Depending on whether an infant is born vaginally or by cesarean section (CS), the maternal impact on the possible transfer of microbes strongly differs. Postnatally, the infant is seeded by the maternal microbiome originating from multiple body niches combined with environmental and food-associated microbes [32]. Next, the microbiota develops by niche-specific differentiation, which is governed by extrinsic host and environmental factors, including breastfeeding and antibiotic usage [33,34].

Intrapartum antibiotics, elective cesarean sections, and low breastfeeding rates have been shown to disturb natural maternal-to-infant microbiome transmission [35]. In the past, different strategies were employed to counteract some of the detrimental effects of these variables. For instance, simulation of the microbial transfer that takes place during vaginal birth by transferring maternal vaginal microbiota to the newborn after a cesarean section by dabbing vaginal fluids to the mouth, nose, or skin of the infant is debated [36]; especially as this practice poses a risk of potentially transferring undiagnosed infections such as group B streptococci, *C. trachomatis*, *N. gonorrhea*, human papilloma virus, group A streptococci, and herpes simplex virus [36]. Another strategy to support the development of the infant microbiome and support the colonization process could be supplementation with various dietary supplements such as probiotics, by dosing either the child-giving person (and relying on vertical transmission) or by dosing the infant directly after birth. In this study, we investigated the administration to infants of a mixture of probiotics including *Bifidobacterium animalis* subsp. *lactis* BB-12 (DSM15954) (BB-12®), *Lacticaseibacillus rhamnosus* LGG (DSM33156) (LGG®), *Lactobacillus acidophilus* LA-5 (DSM13241) (LA-5®), and infant-type *Bifidobacterium longum* subsp. *infantis* Bifin02 (DSM33361) (ISTILOS™) at a relatively low dose in two proof-of-concept single arm clinical studies including equivalent cohorts, performed simultaneously at the same site, either through the child-giving person or directly to the infant. LGG, BB-12, and LA-5 have previously been used in clinical studies investigating perinatal maternal dosing and were found safe and were suggested to have an impact on reduction in the incidence of atopic dermatitis in the offspring [37,38]. LGG and BB-12 have been extensively studied after direct dosing to infants and were found safe, having effects on immune and colic endpoints, respectively [20,21,27,39,40,41]. 

*B. infantis* has been identified as one of the key ‘infant-type’ species which has been related to intestinal and immune maturation and is an abundant member of the gut microbiota during infancy [9,32,42], which makes this subspecies an obvious candidate to include. The aim of these studies was to compare the seeding strategy and relevant endpoints, including recovery of the dosed strains in infant fecal samples, development and metabolic output of the infant gut microbiota, and clinical outcomes. 

## 2. Materials and Methods

### 2.1. General Aspects and Ethical Compliance

The two proof-of-concept studies, maternal seeding (MS) and infant seeding (IS) were conducted at Aarhus University Hospital, Denmark in accordance with the current version of Good Clinical Practice and the Declaration of Helsinki. The study protocols were approved by the local independent ethical committee of Region Midt Denmark (case numbers 1-10-72-15-21 and 1-10-72-14-21). Before any study procedures or collection of data, the General Data Protection Regulation (GDPR) was complied with by written informed consent from the pregnant person and the legal guardians of the participating infant. At Visit 1, the pregnant persons were asked if their perceived gender matches their biological gender or if their perceived gender differs from their biological gender (non-specific). All 47 pregnant persons in the two studies identified themselves as the female gender they were assigned at birth and are henceforth referred to as women or mothers. The infant–mother dyads were recruited from a public midwifery practice between July 2021 and September 2021. The studies were registered at ClinicalTrials.gov (identifier NCT04987593 and NCT04994834) before recruitment.

### 2.2. Participants

For both studies, the mother–infant dyads were included according to the following criteria: The pregnant women were eligible if the pregnancy was uncomplicated, they aimed to give birth vaginally, aimed to breastfeed the infant, and could abstain from the intake of probiotics other than the experimental intervention. Eligible infants were healthy, born at full-term (≥37 and ≤42 weeks of gestational age), with a birth weight between 2500–4500 g, and an APGAR score of at least 7 within the first 10 min of life. Infants were excluded if they had any congenital disorders that could affect their safety or the study outcome or if they were admitted to a neonatal intensive care unit for more than 48 h. 

### 2.3. Study Design

**Figure 1:**
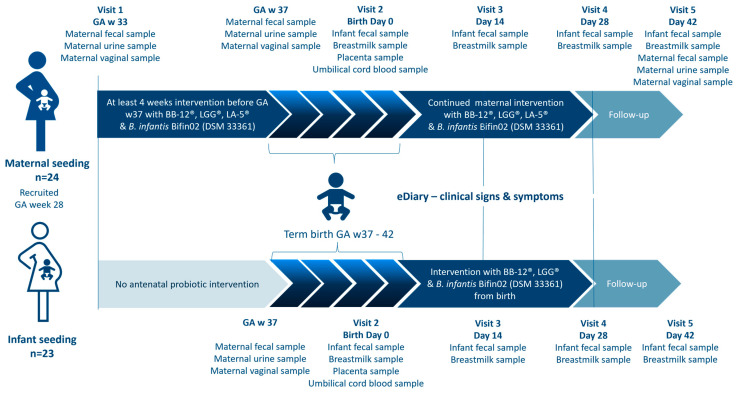
Study flowchart describing the two independent studies, including timepoints for biological sampling.

The two separate open-labelled proof-of-concept studies were conducted at the same site, during the same time, with overall similarity in design and assessments, except for which subject (mother or infant) received the intervention (Figure 1). Due to the aim of the studies and the significant difference in dosing regimens, the studies were designed as separate studies and with an open-labelled design. The studies were conducted in 4 phases: in Phase 1, participants were enrolled, followed by Phase 2, the prenatal phase in which pregnant women participating in the MS study consumed the study products for at least four weeks before gestational week 37. After childbirth, Phase 3, the postnatal intervention period of 28 days (4 weeks) began. Mothers in the MS study continued the intervention and infants in the IS study were given the investigational product as soon as possible within the first 24 h of life. Phase 4 was a two-week follow-up period without any intake of the investigational product (Figure 1).

The screening took place at a public midwifery practice, where the pregnant woman could receive oral and written information about both studies. If eligible and willing to participate, the pregnant woman could choose which study to participate in, whereafter written consent was given on behalf of themselves. During the entire study period, five home visits by the study research midwife were scheduled. Visit 1 consisted of information about the study, obtaining informed consent from the legal parent(s) on behalf of the unborn child, and obtaining maternal information via an electronic case report form (eCRF) (OpenClinica version 4.0, OpenClinica, LLC, Needham, MA, USA). For participants in the MS study, the intervention product was handed out.

Visit 2 was on the day of birth or within 72 h, where the inclusion and exclusion criteria for the infant were checked and infant weight, length, and head circumference were measured. From Visit 2 to Visit 4, the parents completed an electronic diary (electronic patient-reported outcomes) (OpenClinica Participate, OpenClinica, LLC, US). The diary contained selected questions from ROME IV Section A: Infant Gastrointestinal Problems, GERD, and the Amsterdam Stool Form Chart. Some questions were answered daily while others were answered weekly. In Visits 3, 4, and 5, the parents were asked about occurrences of infections, fever, or rash.

Infant growth and thriving were monitored by clinical examinations during Visit 3, 4, and 5. Length and head circumference was measured to the nearest 1 cm by the same non-stretchable measuring tape as the baseline data obtained from the public labor ward. Body weight was measured to the nearest 5.0 g by using an insert on a hanging balance (Kern HDB 10K-2XL). The mean value of two measurements was used. All information collected during the visits was entered into an eCRF by the study research midwife.

In both studies, maternal fecal samples were collected using OMNIgene GUT OM-200 (DNA Genotek Inc., Stittsville, ON, Canada), urine samples were collected using sterile urine sample collection kits (Thermo Fischer Scientific, Waltham, MA, USA), and vaginal samples were collected using OMNIgene Vaginal OMR-130 (DNA Genotek Inc., Canada). Participants in the MS study collected all three biological samples before the intervention (baseline status GA week 33+0) and at day 42. In both studies, the three sample types were all collected at gestational week 37 and at day 42. 

In both studies the legal parent(s) had agreed to collect infant stool samples at four timepoints: the first bowel movements after birth (meconium) and on day 14, 28, and 42 after birth using a fecal sample kit. Additionally, breast milk samples were collected as soon as possible after birth and on day 14, 28, and 42 in both studies. Stool samples and breast milk samples were stored in the household freezer of the participant at a minimum of −18 °C and subsequently transferred to a −80 °C freezer, where they were stored until analysis.

### 2.4. Study Products

In the MS study, the mother consumed the study product from GA week 33+0, to ensure at least four weeks of intervention before term birth (GA week 37–42), and continued consumption for 28 days postnatally followed by a two-week follow-up period without any intake of the study product. The study product used in the MS study consisted of a sachet and a capsule intended to be consumed at the same time. The daily dose was 1 g sachets containing at least 0.1 billion CFU (colony-forming units,) of *Bifidobacterium longum* subsp. *infantis* Bifin02 (DSM33361) and a capsule containing a least 2 billion CFU of *Bifidobacterium animalis* subsp. *lactis* BB-12 (DSM15954), 2 billion CFU of *Lacticaseibacillus rhamnosus* LGG (DSM33156), and 2 billion CFU of *Lactobacillus acidophilus* LA-5 (DSM13241). 

In the IS study, infants were given the study product for 28 days, followed by a two-week follow-up period without any intake of the study product. The first dose was given within 24 (+48) hours after birth. The supplementation continued for 28 days. The study products for the infants were provided as two 1 g sachets containing either freeze-dried culture of at least 0.1 billion CFU of *Bifidobacterium longum* subsp. *infantis* Bifin02 (DSM33361) or at least 1 billon CFU of *Bifidobacterium animalis* subsp. *lactis* BB-12 (DSM15954) and *Lacticaseibacillus rhamnosus* LGG (DSM33156) in total, resulting in a daily dose of at least 1.1 billion CFU. The contents of the two sachets were mixed with 3–5 mL of human breast milk or boiled tap water cooled down to 37 °C before they were given to the infant. 

A subject was considered adherent with study product intake when they consumed at least 80% of the anticipated quantity. The study products were manufactured by Chr. Hansen A/S (Hoersholm, Denmark) and provided in blank containers without any commercial artwork. The strain *Bifidobacterium longum* subsp. *infantis* Bifin02 has previously been studied as BB-02 and will be commercialized under the name ISTILOS™. The other strains are commercially available as BB-12^®^, LGG^®^, and LA-5^®^, trademarks of Chr. Hansen A/S. 

### 2.5. Study Enpoints

#### 2.5.1. Primary Endpoint—Recovery of Dosed Strains in Infant Fecal Samples

The primary endpoint was recovery of the dosed strains, Bifin02, BB-12, LGG, and LA-5, in infant fecal samples after 28 days of intervention, measured via strain-specific quantitative polymerase chain reaction (qPCR) and performed by Baseclear B.V., The Netherlands. The primers used were designed and validated against a relevant panel of genomes that were available at the time of search on the National Center for Biotechnology Information (NCBI) and its internal database (January 2020) (primer sequences are shown in Supplementary Table S1). A dilution series was established for each of the relevant bacteria based on a clean culture to serve as a standard for the calculation of specific targets in experimental samples. All qPCR reactions were performed in 384-well PCR plates (Thermo Fisher Scientific, Waltham, MA, USA) sealed with MicroAmp Optical Adhesive Film (Thermo Fisher Scientific, Waltham, MA, USA) using the Applied Biosystems QuantStudio^TM^ 5 Real-Time PCR system (Thermo Fisher Scientific, Waltham, MA, USA). 

Data was extracted with QuantStudioTM Design & Analysis software v1.4.2, from which targets per gram or ml of raw material were quantified using standard curves. Negative template control (NTC) PCRs were performed alongside each separate amplification without the addition of a template. 

Amplification data were exported from QuantStudioTM Design & Analysis software v1.4.2, followed by the determination of the target quantity per µL of DNA preparation using the standard curves and calculation of the number of targets per g of raw material using the formula below. This method was applied to all qPCR outcomes. Thus, the quantities of specific primer target DNA are expressed as target copies/g material across all sample types.

#### 2.5.2. Fecal Microbiota Composition including the Abundance of Bifidobacteria 

The secondary endpoint was the total relative abundance of bifidobacteria in infant fecal samples after 4 weeks of supplementation and was calculated using 16S rRNA gene amplicon data. Taxonomic profiling using the 16S rRNA gene of the microbiome was performed by BaseClear, The Netherlands, as described below. DNA was extracted from feces using the ZymoBIOMICS 96 MagBead DNA Kit (Zymo Research) and quantified with Quant-iT™ dsDNA Broad-Range Assay Kit (Invitrogen, Waltham, MA, USA) and agarose gel electrophoresis for DNA integrity. The DNA served as a template for PCR amplification of a portion of 16S rRNA genes and subsequent analysis by next generation sequencing using Illumina MiSeq. Amplicons of the V3–V4 regions of 16S rRNA genes, generated by PCR with primers 341F and 785R, which produced a ~630 bp amplicon [43], complemented with standard Illumina adapters. Unique Index Primers were attached to amplicons in each sample with a second PCR cycle. PCR products were purified using Agencourt^©^ AMPure^®^XP (Becker Coulter) and DNA concentration was measured by fluorometric analysis (dsDNA HS kit, Quant-it, Invitrogen). PCR amplicons were equimolarly pooled, followed by sequencing on an Illumina MiSeq with the paired-end 300 cycles protocol and indexing. FASTQ read sequence files were generated using bcl2fastq version 2.20 (Illumina, San Diego, CA, USA). Initial quality assessment was based on data passing Illumina chastity filtering. 

Subsequently, reads containing PhiX control signal were removed using an in-house filtering protocol. In addition, reads containing (partial) adapters were clipped (up to a minimum read length of 50 bp). The second quality assessment was based on the remaining reads using the FASTQC quality control tool version 0.11.8. To generate OTUs (operational taxonomic units), paired-end reads were collapsed into so-called pseudoreads using sequence overlap with USEARCH version 9.2 [44]. Classification of these pseudoreads was performed based on the results of alignment with SNAP version 1.0.23 [45] against the RDP database [46] version 11.5 for bacterial organisms. Next, a table of OTU counts per sample was generated and collapsed into species. Samples containing a low microbial biomass (in our case, the fecal samples taken around birth) are known to be contaminated by extraction kits and laboratory reagents [47]. We first manually removed common contaminating species, genera, and families as reported in [47] and then applied a very conservative prevalence filter to remove spurious OTUs, where an OTU passed the filter if its abundance was at least 0.01% in 2% (4/183) of the samples, which resulted in 287 remaining OTUs in 183 samples. Raw data from sequencing was filtered for Phix signals using bowtie2 (v2.2.6) and trimmed for adapters and a minimum read length of 50 bp with the ea-utils package version 1.04, and samples were screened for chimeras with USEARCH version 9.2. Before creating pseudoreads, reads were trimmed for the 16S primers with cutadapt 2.1 and no additional quality trimming was performed.

#### 2.5.3. Exploratory Endpoints

Gastrointestinal symptoms of infants were assessed daily during the intervention period using questions from ROME IV Diagnostic Questionnaires for Pediatric Functional Gastrointestinal Disorders for Neonates and Toddlers: Parent-Report Form for Neonates Section A Infant Gastrointestinal Problems [48]. Periods and length of periods of inconsolable crying and fussing were measured daily in the ePRO by the parents. Inconsolable crying and fuzzing were defined as periods when the baby cries and fusses without any obvious reason, being impossible to settle down and sooth. Infant stool frequency and consistency were measured using a modified Amsterdam Stool Chart [48]. Reflux was assessed using questions from the Infant Gastroesophageal Reflux Questionnaire Revised (I-GERQ-R). All data were captured using an electronic diary.

Demographics, including information on maternal health and medical history and socioeconomic and household characteristics, were collected at the baseline. Infant baseline characteristics including anthropometry were collected from birth journals. Adverse events, concomitant medication, and healthcare utilization were assessed at each visit and documented in the eCRF. During the COVID-19 pandemic (May–November 2021), the assessments and contacts with subjects were carried out according to relevant national health guidelines.

#### 2.5.4. Biomarkers of Microbial Activity in Feces

The calprotectin (MRP8/MRP14) assay was based on a sandwich enzyme-linked immunosorbent assay (ELISA) (LEGEND MAX™ Human MRP8/14i). The detection range was 3.13–200 ng/mL. The results were normalized to the weight of the original fecal sample.

Endotoxin concentration was analyzed with a specific chromogenic and colorimetric assay (Pierce™ Chromogenic Endotoxin Quant Kit). The detection range was 0.1–1.0 endotoxin units/mL. The results were normalized to the weight of the original fecal sample.

Lactic acid, acetic acid, total SCFA, propionic acid, and butyric acid were derived from the respective phenyl esters using a phenyl chloroformate reagent. Resulting esters were analyzed by Agilent GC-FID. For the quantification, matrix-matched internal standard calibration with butyric-d7 and acetic-d3 acids was used. pH was measured in fecal water samples with a regular pH meter.

### 2.6. Statistical Analysis

All statistical analyses on demographics and clinical outcomes were carried out using SAS v9.4. Windows (SAS Institute Inc., Cary, NC, USA). The safety dataset (SS) consisted of all subjects, whereas the intention-to-treat dataset (ITT) included all mother–infant dyads that completed Visit 2 and met the final eligibility criteria. The per-protocol dataset (PPS) included all mother–infant dyads from the ITT without any major deviations. A *p*-value of less than 0.05 was considered statistically significant. Due to the proof-of-concept and open label design of the two studies, no formal power considerations or sample size calculations were performed. Intending to use one of the studies as a control, we applied the method of stepped rules of thumb for the estimation of the pilot study sample size, which is why a sample size of 25 mother–infant dyads in each study was considered suitable [49].

Baseline demographics, maternal health, medical history, and infant characteristics were analyzed using Mann–Whitney tests for continuous variables and Pearson Chi-square tests for categorical variables. To determine differences in stool consistency between groups, we calculated the area under the curve for each treatment and then a linear mixed model was applied. Inconsolable crying and fussing were analyzed using generalized linear regression analysis.

For gut microbiota data and biomarkers of microbial activity, all analyses were performed in R version 4.2.0 [50] and the R Phyloseq package [51]. To determine which statistical test to employ for all datatypes, we first generated and visually inspected quantile–quantile (QQ plots). If the two datasets did not have the same distribution, we employed a non-parametric Wilcoxon rank-sum test, otherwise a *t*-test for pairwise comparisons was used. Specifically, to test for differences in the relative abundance of species between two groups, due to the compositionality [52] of the 16S rRNA gene amplicon data, we employed center log ratio transformation (CLR) [53] and corrected for multiple testing using the Benjamini–Hochberg (BH) procedure. An uncorrected P-value or corrected q-value of <0.05 was considered statistically significant.

## 3. Results

### 3.1. Subject Disposition and Characteristics

**Figure 2:**
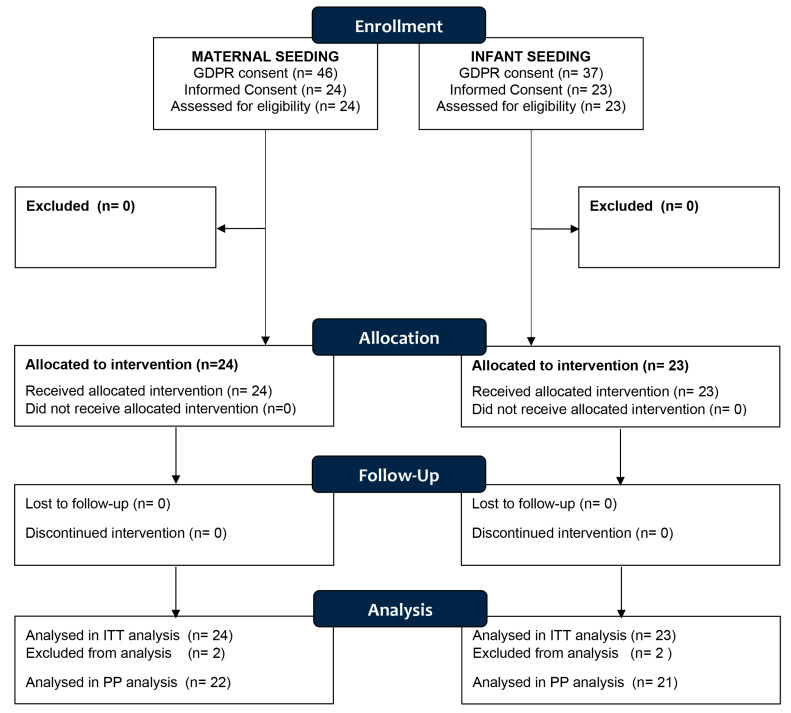
CONSORT diagram showing the disposition of subjects.

Eighty-three pregnant women were screened for eligibility for the two studies at Aarhus University Hospital and provided with both oral and written information about the two studies. They all signed the GDPR consent, which allowed the research midwife to send the informed consent form via an e-mail link. In total, 47 pregnant subjects volunteered to join one of the studies: 24 for the MS study and 23 for the IS study (Figure 2).

#### 3.1.1. Maternal Characteristics

Maternal age at enrollment, ethnicity, body mass index (BMI), activity level before pregnancy, smoking, and dietary habits did not differ between the two study groups (Table 1). An equal distribution in both intra- and inter-intervention groups was observed regarding nulliparous and multiparous women (e.g., 12 (52.2%) vs. 11 (47.8%) in the IS group and 13 (54.2%) vs. 11 (45.8%) in the MS group, respectively). Four women in the IS group and three women in MS group were screened for Group B *Streptococcus* (GBS) within 24 h before birth due to the risk of infection. Three women (two women from the IS group and one woman from the MS group) tested positive and antibiotic treatment was initiated. There was no difference in mean hours from the pre-labor rupture of membranes (PROM) to birth between the groups or between the number of vaginal explorations before PROM/labor and the volume of postpartum hemorrhages. Maternal baseline and clinical characteristics are presented in Table 1.

#### 3.1.2. Infant Characteristics 

All infants were term born (GA week 37–42) except for one in the MS group, born in GA week 34+1. One infant in each group was born by an emergency cesarean section. Gender distribution, birth weight, length, and head circumference were not different between the infants in the two studies. Two infants from the IS group and one from the MS group were exposed to antibiotics postnatally due to a suspected infection. Skin-to-skin contact was established for all infants within 24 h, and no infants had a bath within 24 h after birth. An intramuscular K-vitamin injection (1 mg phytomenadione) was given to all infants within the first two hours after birth in compliance with the recommendation of the Danish National Board of Health. 

All infants were put to the breast immediately after birth and received colostrum within the first hours after birth. They were predominantly breastfed from birth throughout the study. Five (21.7%) infants in the IS group and four (16.7%) infants in the MS group were supplemented with infant formula during the first 24 h after birth due to various reasons. The given volume of infant formula ranged from below 20 mL to 80 mL, did not contain pre- or probiotics, and was equally distributed in the two groups. Twenty-one (91.3%) of the infants in the IS group were exclusively breastfed throughout the study, compared to twenty-three (95.8%) infants in the MS study. The number of infants who received partially mixed feeding during the first two weeks after birth was equal in both groups (three in the IS group vs. four in the MS group). In compliance with Danish dietary recommendations, all infants received a D-vitamin supplement as a drop suspension (without probiotics) from two weeks of age. Infant baseline characteristics are presented in Table 2. 

### 3.2. Primary Outcome: Recovery of Dosed Strains in Infant Fecal Samples

**Figure 3:**
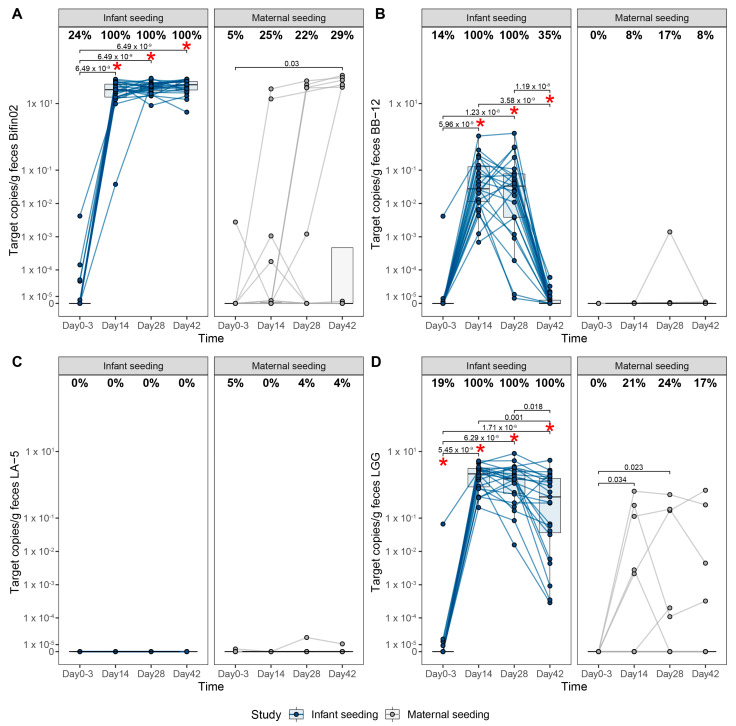
Recovery of the dosed strains by qPCR. Prevalence (% of positive subjects) and abundance of target copies/g feces of (**A**) Bifin02, (**B**) BB-12, (**C**) LA-5, and (**D**) LGG. All *p*-values are calculated using pairwise group comparison based on Wilcoxon rank-sum tests. Within the study, *p*-values are shown as numbers, whereas a red asterisk depicts a significant difference (*p* < 0.05) between the two dosing regiments at that specific time point.

Both maternal and infant supplementation with the probiotic strains Bifin02, BB-12, and LGG resulted in the recovery of these strains from infant fecal samples (Figure 3). 

In the IS group, the recovery (% of infants with a detected strain) of Bifin02 was identified in 100% of the infants from 14 days onwards, compared to 25%, 22%, and 29%, for day 14, day 28, and day 42 in the MS group, respectively. Similar patterns were observed for LGG: 100% recovery in the IS group compared to 21%, 24%, and 17%, respectively, in the MS group. In the IS group, recovery of BB-12 was 100% on day 14 and day 28 but declined to 35% on day 42, compared to 8%, 17%, and 8% on day 14, 28, and 42, respectively, in the MS group. Three infants in the IS group received the first dose of the study product before it was possible to collect the first fecal sample; therefore, those infants had all three dosed strains present in the first fecal sample. 

Although in the IS group, the prevalence of Bifin02 was already 100% from 14 days onwards, the mean abundance slightly increased throughout (Figure 3A). The intra-individual trajectories indicated that within subjects, the abundance of Bifin02 already stabilized after 14 days of dosing, remained stable throughout the dosing period, and continued at least 14 days after cessation of dosing at day 42. In the MS group, colonization with Bifin02 was delayed compared to the IS group; however, the infants from whom the strain was recovered showed colonization (i.e., stable abundance throughout the dosing period and an increase in abundance 14 days after cessation of dosing). BB-12 was detected at a higher percentage in the IS group compared to the MS group during intervention. After dosing cessation, BB-12 was only detected in 35% of the infants, which suggests that the presence of BB-12 was transient. The same pattern was observed in the infants in the MS group but to a lower extent and in a very low quantity (Figure 3B). 

In the IS group, the trajectory LGG showed that the quantity of this strain decreased after dosing was stopped. Nevertheless, its prevalence was still 100% 14 days after the cessation of dosing, indicating that, at least during the study period, this strain colonized all infants (Figure 3D). LA-5 was only dosed in the MS group and was recovered on day 28 and day 42 from one infant (Figure 3C). Interestingly, the abundance of Bifin02 was orders of magnitude greater than that of LGG and BB-12, respectively, despite the lower daily dose of Bifin02. 

Abundances of the dosed strains were also measured in different biological specimen samples from the mother, including fecal, urine, vaginal, and breastmilk samples. The strains dosed in the IS study were detected at all four timepoints in up to 60% of the breastmilk samples, in contrast to the MS study, where only one to two subjects had the presence of the dosed strains (Table S1).

The dosed strains were detected in the majority of the maternal fecal samples in the MS group (Table S2) at GA week 37. Samples from the placenta, umbilical cord, and umbilical cord blood were also analyzed, with no detection of the dosed strains at all. Results from the strain-specific qPCR are presented in Supplementary Tables S1–S5. 

Remarkably, BB-12 and LA-5 were also recovered from fecal samples from five mothers in the IS group who had inadvertently used a common probiotic fermented milk product that includes these strains. Nevertheless, their intake was limited to a few occasions. 

### 3.3. Relative Abundance of Total Bifidobacteria and Microbiota Composition of Infant Fecal Samples

**Figure 4:**
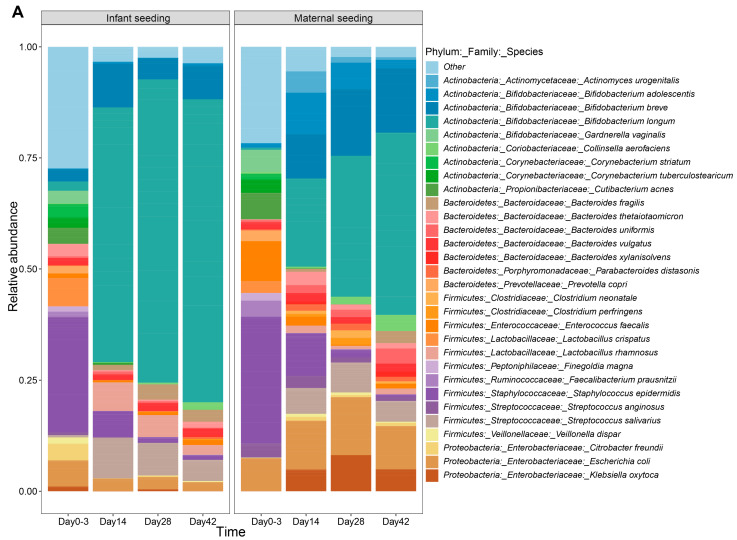

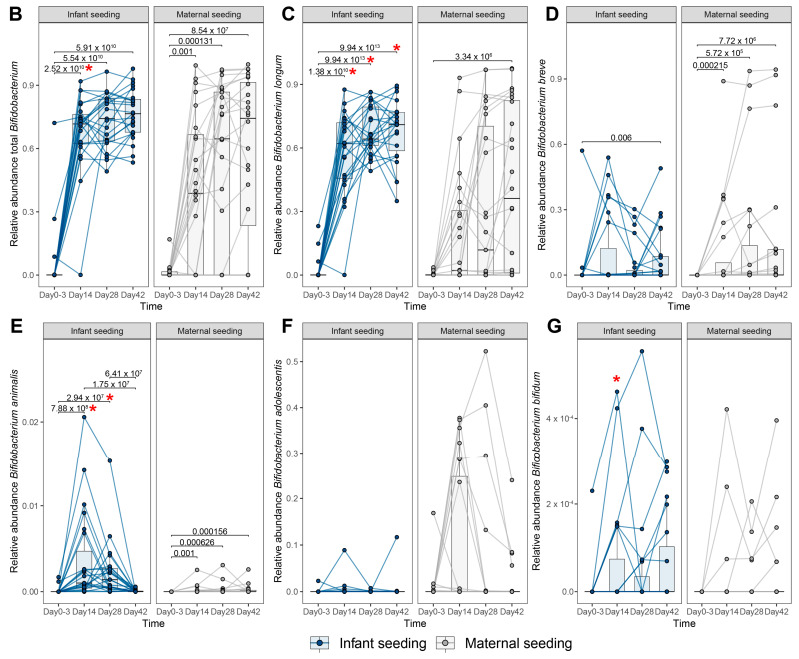
Microbial composition of infant fecal samples from the infant seeding versus the maternal seeding study. (**A**) Relative abundance of the top 30 species, whereas the rest of the species are summarized as “other”. (**B**) Relative abundance of total bifidobacteria. (**C**–**G**) Relative abundance of separate *Bifidobacterium* species. All *p*-values were calculated using pairwise group comparison based on Wilcoxon rank-sum tests. Study *p*-values are shown within study facets while a red asterisk depicts a significant difference (*p* < 0.05) between the two dosing regiments at that timepoint. The higher group contains the asterisk.

Microbiota profiling was successful for 183 samples and yielded a mean of 12812 ± SD 2778 reads per sample. In the IS group, the relative abundance of *Bifidobacterium* was higher compared to that at birth at all timepoints (q = 3.6 × 10^−7^, 6.8 × 10^−11^, 3.0 × 10^−8^ at day 14, 28, and 42, respectively) (Figure 4B). The difference in *Bifidobacterium* abundance between the two groups diminished over time. The lower abundance of *B. longum* (*infantis*) in the MS group was compensated for by a higher abundance of *B. breve* and *B. adolescentis* OTUs (operational taxonomic units) compared to the IS group (Figure 4A,B). Altogether, the increase in the genus *Bifidobacterium* in the MS group was more gradual compared to the IS group and did not become significant until day 28 and onwards (q = 4.4 × 10^−3^, 7.1 × 10^−4^, at day 28 and 42, respectively). 

Although *Bifidobacterium* increased more from birth to day 14 in the IS group compared to the MS group, the total *Bifidobacterium* abundance on day 14 was not significantly different between the two interventions due to large inter-individual differences and correction for multiple testing (*p* = 0.004, q = 0.18) (Figure 4A,B).

In the first fecal sample, collected soon after birth, the microbiota contained bacteria typically associated with the birth canal, vagina, and skin, including *Lactobacillaceae*, *Staphylococcaceae*, and *Enterobacteriaceae*. These were all aerobic and aerotolerant/microaerophilic species, and the compositions were similar between studies.

In the IS group, compositional changes were mainly due to large increases in bifidobacteria, lactobacilli, and streptococci and concomitant decreases in typical skin bacteria such as corynebacteria, cutibacteria, and staphylococci, as well as several proteobacteria. Although the most dramatic effects occurred from birth to day 14, these dynamics further evolved even after the cessation of dosing. In contrast, these dynamics were delayed by 14 days or more in the MS group, largely because some infants still had bifidobacteria below the detection threshold (while the IS infants were mostly above 40%). The aforementioned large increases in bifidobacteria, lactobacilli, and streptococci and concomitant decreases in typical skin bacteria such as corynebacteria, cutibacteria, and staphylococci, as well as several proteobacteria, were not observed in the MS group until day 28.

### 3.4. Infant Gastrointestinal Signs and Symptoms

**Figure 5:**
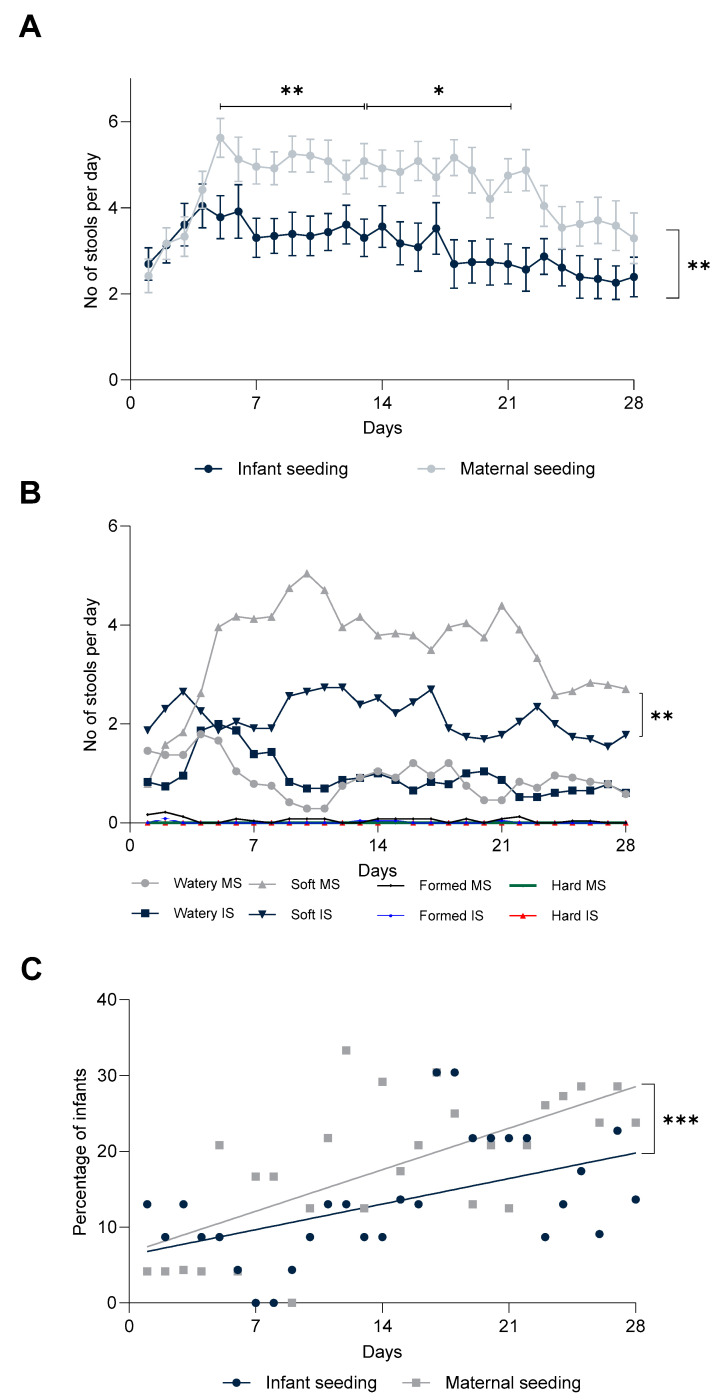
(**A**) Mean daily stool frequency. Significance was calculated using the Mann–Whitney U test. (**B**) Number of mean daily stools of the four different consistencies. Significance calculated using the Mann–Whitney U-test. (**C**) Percentage of infants with inconsolable crying and fussing in the two groups. *p*- value and analysis is based on the residual variance method (SAS). IS = Infant seeding group, MS = maternal seeding group. (* *p* < 0.05, ** *p* < 0.05, *** *p* < 0.001).

Infant stool frequency (number of bowel movements per day) during the first week after birth was similar in the IS and MS groups (mean 3.9 ± 1.4 and 3.4 ± 1.6 stools per day, respectively) (*p* = 0.3151). From day 14 onward, mean stool frequency was significantly lower in the IS group (*p* = 0.0058) compared to the MS group. Additionally, the IS group passed fewer stools per day (at day 14 *p* = 0.0027 and at day 28 *p* = 0.0231) and in total over the 28 days of intervention calculated by AUC (area under curve), *p* = 0.0052 (Figure 5A).

A significant difference in stool consistency was also observed between the two dosing regimens, with MS infants passing softer stools more frequently than the infants in the IS group during week 2, 3, and 4 (*p* < 0.001, *p* < 0.001, *p* = 0.045). The mean number of watery, formed, and hard stools was similar in both groups throughout the intervention (Figure 5B).

Occurrences of episodes of inconsolable crying and fussing were measured daily, and we observed a significant progression in the percentage of inconsolable infants in the MS group. The slope of progression for inconsolable crying and fussing during the intervention period was significantly less steep in IS infants compared to MS infants (*p* < 0.0001) (Figure 5C).

No differences were observed between the two groups regarding gastrointestinal signs and symptoms (infants showing signs of struggle, gruntiing, or crying before a bowel movement, straining at defecation or flatulence).

### 3.5. Fecal Biomarkers of Microbial Activity

**Figure 6:**
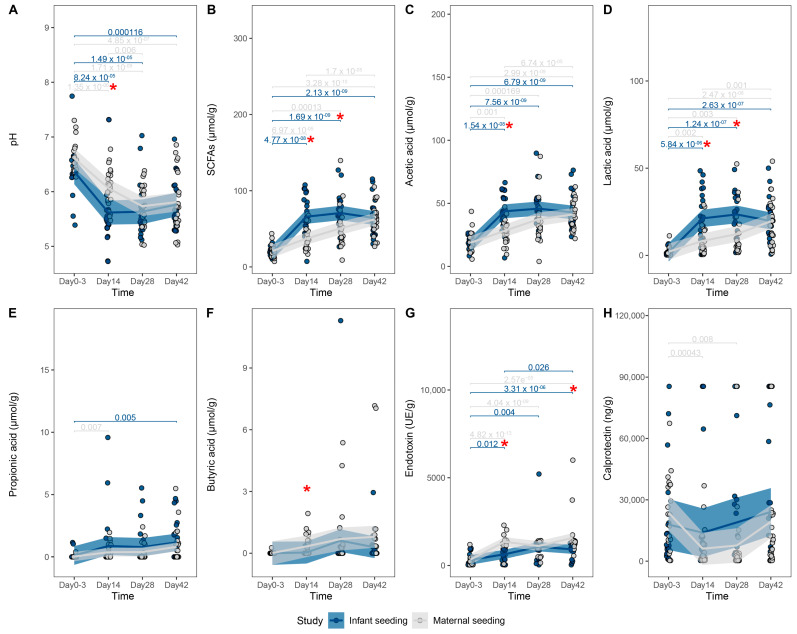
Comparison of fecal markers of microbial activity between the two dosing regimens, including (**A**) pH, (B) Short Chain Fatty Acid (SCFA), (**C**) Acetic acid, (**D**) Lactic acid, (**E**) Propionic acid, (**F**) Butyric acid, (**G**) Endotoxin and (**H**) Calprotectin All *p*-values are calculated using pairwise group comparison based on Wilcoxon rank-sum or *t*-tests, depending on data distribution. Study *p*-values are colored by study group, while a red asterisk depicts a significant difference (*p* < 0.05) between the two dosing regimens at that timepoint. The outcomes of both treatments are summarized and smoothed using non-parametric locally estimated scatterplot smoothing (LOESS), with the shaded area representing a 95% confidence interval.

Fecal pH was significantly lower at day 14 in the IS group compared to the MS group (5.6 ± 0.13 vs. 6.1 ± 0.08, respectively, *p* = 0.0046) (Figure 6A). 

At day 14 after birth, the levels of total short chain fatty acids (SCFAs) (65.9 ± 5.8 µmol/g vs. 37.6 ± 3.4 µmol/g, *p* < 0.0001) were higher in the IS group compared to the MS group (Figure 6B). A significant difference between the two groups was also observed for acetic acids at day 14 (*p* < 0.05) (Figure 6C). The same pattern was observed for lactic acids even though this was significant at both day 14 (*p* < 0.05) and day 28 (*p* < 0.05) (Figure 6D). No statistical significance was observed for propionic acids between the two groups; however, a significant increase in propionic acids was observed in the IS group at the end of study day 42 (Figure 6E). Butyrate was higher in the MS group on day 14 compared to the IS group (0.324 ± 0.112 µmol/g vs. 0.0367 ± 0.0254 µmol/g, respectively, *p* = 0.012) (Figure 6F). 

Fecal endotoxin levels were lower in the IS group on day 14 (640.6 ± 92,0 vs. 1365.6 ± 84.3 UE/g, *p* < 0.0001) and day 42 (918.1 ± 82.5 vs. 1601.7 ± 242.1 UE/g, *p* = 0.01) compared to fecal levels from MS infants (Figure 6G). The measured amount of calprotectin was not different at any timepoint (Figure 6H).

### 3.6. Correlations between Various Endpoints and Bacterial Species in Infant Fecal Samples

**Figure 7:**
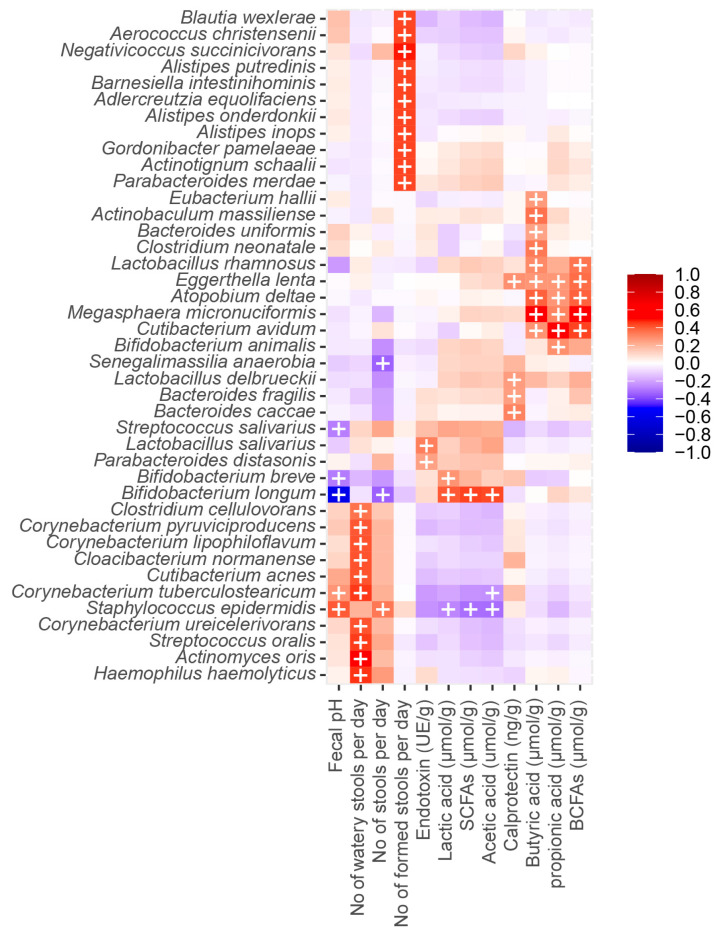
Correlations of microbiota with the clinical endpoints of stool consistency/frequency, crying and fussing, and other markers of microbial activity in feces. The heatmap is colored by strength of the Pearson correlation and “+” signs depict significant associations after correcting for multiple testing (q < 0.05) using the Benjamini–Hochberg procedure.

*Bifidobacterium longum* was significantly inversely correlated with fecal pH and stool frequency and positively correlated with lactic acids, acetic acid, and SCFA. *Bifidobacterium animalis* was significantly correlated to propionic acid, whereas a non-significant inverse correlation was observed with stool frequency. *Lactobacillus rhamnosus* was significantly correlated with the presence of butyric acid and branch-chained fatty acids (BCFAs) (Figure 7). The number of watery stools per day was significantly correlated with the presence of species from *Clostridium*, *Corynebacterium*, *Cloacibacterium*, *Cutibacterium, Staphylococcus*, *Streptococcus*, *Actinomyces*, and *Haemophilus*, whereas the number of formed stools was significantly correlated with the presence of species from *Blautia*, *Aerococcus*, *Negativicoccus*, *Alistipes*, *Barnesiella*, *Adlercreutzia*, *Alistepes*, *Gordonibacter*, *Actinobaculum*, and *Parabacteroides* (Figure 7).

### 3.7. Safety, Tolerability, and Adverse Events

There were no significant differences in mean infant weight, length, or head circumference between the two intervention groups during the study, and all infants were thriving. No differences were observed in the safety and tolerability assessments either (Supplementary Table S6). 

No adverse events were reported for the duration of both interventions. One infant in the IS group reported wheezing and problems with eating during the first two weeks after birth. This was assessed by the research midwife as not being related to intake of study products.

The presence of Group B *Streptococcus* was diagnosed during labor in two mothers from the IS group and one mother from the MS group. One mother from the MS group reported a bladder infection during the follow-up period. All four cases were treated and not related to the study product intake. 

## 4. Discussion

These two studies are the first of their kind to compare the recovery, route of transfer, and potential colonization of probiotic strains after two different infant dosing regimens. Infants were either dosed directly after birth or indirectly via the mothers during the last months of pregnancy and the first 28 days after birth, thus relying on vertical bacterial transmission during or soon after birth for colonization. It is noteworthy to mention that dosing the infants directly from birth does not raise any safety concerns.

Transfer of bacteria from mother to infant during vaginal birth is proposed to be conveyed through vertical transmission of microbes from the delivery environment through maternal body sites such as the skin, vagina, and feces [35,54,55]. Compared to CS-born infants, the estimated impact of the maternal fecal microbiota on the microbiota of vaginally born infants was reported to be significantly higher (week 1, 16.6% vs. 4.5%; week 2, 21.7% vs. 10.3%). This higher fraction was associated with a higher transfer rate of *Bifidobacterium* spp. [35]. Our data are strikingly concordant with these findings, where fecal recovery of Bifin02 was observed in 25%, 22%, and 29% of MS infants at day 14, day 28, and day 42, respectively. Our observations support the sterile womb hypothesis [31], as we did not recover the dosed strains in samples from the maternal vagina, urine, placenta, or umbilical cord. In contrast, it seems that the dosed strains found in fecal samples from the MS infants were transmitted from the maternal feces mainly, which is in concordance with increased actinobacteria abundancy from the first to the third trimester in pregnant women [56]. Interestingly, we detected the dosed strains in breastmilk samples from mothers from the IS study, where only the infants were consuming the probiotics. This suggests a cross-contamination from the infant’s oral cavity during breastfeeding to the breast surroundings. This has also been described by others, where the milk microbiome has been found to be more similar to the infant oral microbiome than the infant fecal microbiome [57]. In contrast, the dosed strains were only detected in a few breastmilk samples from the MS study.

Importantly, infants directly seeded with Bifin02 maintained it over time. The persistence of Bifin02 after termination of the intervention and the stable trajectories of the strain in individual infants, as observed in both treatment groups, strongly suggests engraftment of the strain even after being dosed at a relatively modest daily dose of 0.1 billion CFU. In several previous studies, strain *B. infantis* EVC001 was dosed to infants in significantly higher doses of 18 billion CFU or more, which led to dominance of *Bifidobacteriaceae* (86% mean relative abundance at day 14 in vaginally born infants dosed with 18 billion CFU of *B. infantis* EVC001 compared to only 36% in the control group) [58,59]. 

In contrast, our findings suggest that Bifin02 engrafts without complete dominance and enhanced colonization of other bifidobacteria. These changes are supported by the observed alterations in the metabolic output of the microbiome on day 14, resulting in a lower fecal pH and endotoxin level and increased acetate, butyrate, and total SCFA production in the IS infants. Butyrate, a SCFA, has been shown to exert immunomodulatory functions by the inhibition of histone deacetylases (HDAC) and the activation of G protein-coupled receptors (GPCR) on epithelial and immune cells. Overall, SCFA supports the development of a healthy gastrointestinal tract, modulates immune responses, and promotes the growth of colonocytes during infancy [60].

The other dosed strains, mainly BB-12 and LGG, were detectable in the infant fecal samples, though at a lower level and frequency in the fecal samples from MS-dosed infants compared to directly dosed infants. Lactobacilli are frequently found in the infant microbiome [32,61], and in our IS study, the prevalence of LGG was 100% even after cessation, implying colonization. However, the abundance of LGG slowly declined over time, suggesting only transient colonization that may cease over time when dosing has come to an end. In contrast, the probiotic strain BB-12 did not display the same degree of colonization. Nonetheless, it has been repeatedly shown that BB-12 both alone and in combination with other strains can bring important health benefits to both preterm and term infants [20,21,62,63]. 

Infancy is characterized by large variations in stool frequency and consistency, both between and within infants [64]. In a cohort of 600 healthy Dutch infants, breastfed infants showed an average daily defecation frequency of 3.65 times per day in the neonatal period, which decreased significantly during the first 3 months to 1.88 times per day, whereas no significant changes were observed in infants fed with standard formula or mixed feeding. At every age, both the average and the range of defecation frequency of breastfed infants were higher than those of infants receiving formula, and breastfed infants had softer feces than formula-fed infants [65]. The higher defecation frequency of breastfed infants was also observed in a study by Moretti et al. [64], who compared daily stool frequency and observed a significantly higher daily stool frequency in breastfed infants compared to formula-fed infants during the first month after birth (4.9 ± 1.7 vs. 2.3 ± 1.6, respectively.) These data are in concordance with the defecation frequency observed in our cohort of predominantly breastfed infants; however, a difference in stool frequency between the two dosing regimens was observed, where the infants dosed directly passed fewer stools per day during the first 28 days of intervention compared with infants dosed through the mother. The interplay between the feeding mode, the microbiota, the microbial metabolites, and stool frequency and consistency is still a field which needs further exploration. As the feeding mode is the same between both treatment groups, the microbe-related signatures such as fecal pH, SCFAs such as butyrate, and acetate concentrations may play a role in and impact bowel habits [66].

Previous studies have shown an effect of BB-12 on reducing crying and fussing in infants with colic [20,21]. Infant colic, manifested as episodes of inconsolable crying and fussing, has been associated with a higher abundance of proteobacteria and a lower abundance of bifidobacteria [21,22,26,27]. The difference between the study groups with respect to fewer infants in the IS group expressing inconsolable crying and fussing could be explained by an early bifidobacteria-dominated microbiota leading to the maturation of the gastrointestinal tract. Several mechanisms have been proposed with respect to the effects of *Bifidobacterium* species on intestinal maturation, including skewing T-cell proliferation, leading to a more anti-inflammatory state and modulation of barrier integrity [9].

The impact of the neonatal microbiome on clinical signs and symptoms from the gastrointestinal tract, such as crying and fussing, and on the normal healthy immune maturation underlines the importance of this period. It is believed that early infancy is a window of opportunity in which the gut microbiota is easier to modulate [67] and in which infant-type bifidobacteria are suggested to play a vital role in the maturation of the gastrointestinal tract and in educating the neonatal immune system [9], which may lay the foundation of health in later life. Studies have reported increases in species of Proteobacteria, and some studies reported a decrease in lactobacilli and bifidobacteria in infants with colic [68]. We observed a change in microbiome towards bifidobacteria and lactobacilli enrichment in the same cohort which showed less inconsolable crying and fussing. This enables the hypothesis that an association exists between an increased abundance of bifidobacteria, fewer bowel movements, and less crying and fussing in the neonatal phase.

All infants included in our two studies were predominantly breastfed, the only difference was the intervention. It has been shown that breastfed infants have a higher abundance of lactate and acetate metabolites compared to bottle-fed children [69]. These findings might be explained by a difference in microbial composition, and correspond to our observations of a significant difference in SCFAs, acetate, and lactic acid at day 14 and 28, which could be explained by the significant difference in Bifin02, a *Bifidobacterium longum* subspecies *infantis* known for its prodigious capacity to digest and consume human milk oligosaccharide structures due to the result of a unique cluster of bacterial genes encoding an array of glycosidases and oligosaccharide transporters not found in other bacterial species [70]

The strength of our studies is the high compliance and adherence to the study procedures, which resulted in robust and detailed data from both mothers and infants. We also acknowledge several limitations. Randomized trials are considered the gold standard when assessing efficacy, and the current studies were conducted as open-label studies with no control groups. It was an active choice not to include control groups due to the proof-of-concept design and the ambition to use each study group as a control for the other. The use of a placebo group in this uniquely vulnerable period of life should always be considered carefully and should always be justified by a positive risk–benefit analysis. Another limitation is the use of 16S rRNA, which does not provide the most accurate and reliable profiling of bacterial subspecies. However, complementary analysis and the use of strain-specific qPCR confirmed our conclusions regarding the presence of the dosed strains. To confirm the potential positive influence of early seeding on health later in life, follow-up studies are warranted.

## 5. Conclusions

Dosing infants directly or indirectly via maternal consumption with *Bifidobacterium longum* subsp. *infantis* Bifin02 (DSM33361), *Bifidobacterium animalis* subsp. *lactis* BB-12 (DSM15954), and *Lacticaseibacillus rhamnosus* LGG (DSM33156) was safe and well tolerated. Dosing infants directly after birth resulted in 100% of the infants being colonized with Bifin02 after 14 days, as opposed to 25% after 28 days of dosing the mothers during the last months of pregnancy and the first 28 days after birth. Thus, maternal oral supplementation with these strains supports a direct strain transfer from mother-to-child through fecal transmission. Bifin02 colonization was achieved with a moderate dose of only 10^8^ CFU (0.1 billion). Additionally, dosing infants directly modulated the microbiota, leading to a faster transition towards *Bifidobacterium* domination, which was correlated with a lower defecation frequency and a slower increase in the incidence of crying and fussing.

Our observations support safe and targeted food supplementation both pre- and postnatally to the mother and directly to the newborns to ensure a healthy gut microbiome for the infant child.

## Figures and Tables

**Table 1:** Maternal baseline demographics and clinical characteristics.

Maternal Characteristics	Infant Seeding (*n* = 23)	Maternal Seeding (*n* = 24)	*p*-Value
Maternal age at birth	31.0 ± 0.8	30.1 ± 3.7	0.4388
Pre-pregnancy BMI	22.4 ± 2.1	22.7 ± 2.9	0.8489
Non-nicotine use during, *n* (%)	23 (100.0)	24 (100.0)	0.5100
Maternal Ethnicity, *n* (%)			0.5100
Caucasian	15 (65.2)	21 (87.5)	
Asian	1 (4.3)	0	
Mixed	6 (26.2)	2 (8.3)	
Other	1 (4.30)	1 (4.2)	
Highest level of education, *n* (%)			0.2417
Upper secondary education	0	2 (8.3)	
Short-cycle tertiary education	0	2 (8.3)	
Bachelor’s or equivalent level	8 (34.8)	7 (29.2)	
Master’s or equivalent level	15 (65.2)	13 (54.2)	
Exercised during pregnancy, *n* (%)	20 (87.0)	24 (100.0)	0.2756
Level of stress, *n* (%)			0.5773
Low	18 (78.3)	17 (70.8)	
Medium	5 (21.7)	6 (25.0)	
High	0	1 (4.2)	
Children (<18) in the household, *n* (%)			0.5773
0	12 (52.2)	14 (58.3)	
1	6 (26.1)	8 (33.3)	
2	4 (17.4)	2 (8.3)	
3	1 (4.3)	0	
Domestic pets, *n* (%)	6 (26.1)	2 (8.3)	0.3571
Dog	5 (83.3)	0	
Cat	1 (16.7)	2 (100.0)	

**Table 2:** Baseline demographics and clinical characteristics of the infants in the two studies.

Infant Characteristics	Infant Seeding (*n* = 23)	Maternal Seeding (*n* = 24)	*p*-Value
Infant Gender			0.8815
Male	12 (52.2)	12 (50.0)	
Female	11 (47.8)	12 (50.0)	
Gestational age at birth, weeks	39.7 ± 1.0 (37.0–41.0)	39.7 ± 1.7 (34.0–42+0)	0.5470
Mode of birth, *n* (%)			0.2149
Vaginal, unassisted	22 (95.7)	23 (95.8)	
Assisted vaginal	0	0	
Emergency Cesarean section	1 (4.3)	1 (4.2)	
Birth weight, g	3692 ± 549.2	3516 ± 497.7	0.4108
Birth length, cm	52.3 ± 2.0	51.7 ± 2.0	0.3677
Birth head circumference, cm	34.8 ± 1.2	34.4 ± 1.6	0.2683
Apgar Score >7/5 min, *n* (%)	23 (100.0)	24 (100.0)	
Oral or IV antibiotic used postnatally, *n* (%)	2 (8.7)	1 (4.2)	0.5255
Intake of colostrum, *n* (%)	23 (100.0)	24 (100.0)	0.3224
Infant formula during the first 24 h, *n* (%)	5 (21.7)	4 (16.7)	0.6586
Bath during the first 24 h, *n* (%)	0	0	

## Data Availability

The data presented in this study will be available upon request.

## Supplementary Materials

The following supporting information can be downloaded at: https://www.mdpi.com/article/10.3390/nu15184000/s1, Table S1: Strain-specific primer set; Table S2: Strain-specific qPCR data in maternal breastmilk samples; TableS3: Strain-specific qPCR data in maternal fecal samples; Table S4: Strain-specific qPCR data in vaginal samples; Table S5: Strain-specific qPCR data in maternal urine samples; Table S6: Strain-specific qPCR data in placenta, umbilical cord and umbilical cord blood samples; Table S7: Infant growth and tolerability assessments.
