# Supplementary material for: Seeding the Infant Gut in Early Life—Effects of Maternal and Infant Seeding with Probiotics on Strain Transfer, Microbiota, and Gastrointestinal Symptoms in Healthy Breastfed Infants
Source: Nutrients. 2023 Sep 15;15(18):4000. doi: 10.3390/nu15184000 (PMC10538230; doi:10.3390/nu15184000)
Supplement: Supplementary file 1 [file nutrients-15-04000-s001.zip › nutrients-2528374-supplementary.pdf]

Supplementary

Table S1. Primers for targeting Bifin02, BB-12, LGG and LA-5

	**Primer name**	**Sequence 5'-3'**	**Amplicon size**	**Target strain**
**1**	BB-02#Assay02_F	CTTTGAATCCGTCGATAAAACA		Bifin02
**2**	BB-02#Assay02_R	TATATCGTCGCGGCATTCTT		
**3**	9689_c12_F1	ACGGATTCGAACGGTCTTC	102	BB-12
**4**	9689_c12_R1	CGTGTCTCCCTGACCTATCG		
**5**	11994#Assay2_F	CTCTGGCGTATTACCATCCAA	60	LGG
**6**	11994#Assay2_R	GAGATTCCATCCCAGACCAA		
**7**	8866_c8_1F	ACGGCGTTGTTCCCACTTAT	69	LA-5
**8**	8866_c8_1R	TCACCTGTTCGCCCACTTAC		

Table S2. Strain-specific qPCR data in maternal breastmilk samples, no of subjects (quantity target per g material)

	Infant seeding group				Maternal seeding group			
	Day 0	Day 14	Day 28	Day 42	Day 0	Day 14	Day 28	Day 42
Bifin02	11 (6,10E-05 ±3,74E-05)	13 (1,55E-04 ±1,05E-04)	11 (1,36E-05 ±4,77E-06)	10 (1,24E-05 ±6,38E-06)	0	3 (5,70E-06± 4,32E-06)	2 (2,44E-05 ±2,23E-05)	2 (3,59E-05 ±2,49E-05)
BB-12	14 (1,75E-04 ±1,18E-04)	8 (1,42E-04 ±1,32E-04)	9 (4,22E-06 ±2,03E-06)	5 (8,24E-07 ±4,50E-07)	0	0	1 (1,77E-06)	1 (3,95E-08)
LGG	13 (3,57E-04 ±2,31E-04)	12 (3,36E-04 ±2,93E-04	4 (1,58E-05 ±9,38E-06)	4 (1,96E-06 ±1,04E-06)	0	0	1 (2,23E-05)	0
LA-5	0	0	0	0	2 (1,44E-06± 1,32E-06)	1 (5,54E-06)	1 (5,54E-06)	0

Table S3. Strain-specific qPCR data in maternal fecal samples, no of subjects (mean quantity target per g material)

	Infant seeding group					Maternal seeding group					
	GA 37	Day 0	Day 14	Day 28	Day 42	GA 33	GA 37	Day 0	Day 14	Day 28	Day 42
Bifin02	0	-	-	-	-	1 (0,0000125)	18 (0.0000639)	-	-	-	2 (0.000000023)
BB-12	5 (1,94E-03±1,92E-03	-	-	-	-	5 (1,62E-03 ±1,53E-03)	21 (2,25E-02 ±7,76E-03)	-	-	-	5 (3,52E-04 ±3,51E-04)
LGG	1 (2,08E-05)	-	-	-	-		Antal? 0,001273009±0,00064638				
LA-5	5 1,30E-02 ±4,15E-02)	-	-	-	-	6 (7,49E-04 ±4,73E-04)	21 (4,72E-03 ±2,41E-03)	-	-	-	2 (3,71E-02 ±3,69E-02)

Table S4. Strain-specific qPCR data in vaginal samples, no of subjects (quantity target per g material)

	Infant seeding group					Maternal seeding group					
	GA 37	Day 0	Day 14	Day 28	Day 42	GA 33	GA 37	Day 0	Day 14	Day 28	Day 42
Bifin02	1 (0,00641)	-	-	-	-	0	0	-	-	-	0
BB-12	0	-	-	-	-	0	1 (1,26E-02)	-	-	-	0
LGG	0	-	-	-	-	0	1 (3,26E-07)	-	-	-	0
LA-5	2 (3,68E-03 ±3,20E-04)	-	-	-	-	5 (5,94E-04 ±4,04E-04)	4 (1,96E-02 ±1,66E-02)	-	-	-	0

Table S5. Strain-specific qPCR data in maternal urine samples, no of subjects (quantity target per g material)

	Infant seeding group					Maternal seeding group					
	GA 37	Day 0	Day 14	Day 28	Day 42	GA 33	GA 37	Day 0	Day 14	Day 28	Day 42
Bifin02	1 (0.00104)	-	-	-	-	0	1 (0.0353)	-	-	-	0
BB-12	0	-	-	-	-	1 (2,66E-03)	1 (9,47E-04)	-	-	-	0
LGG	0	-	-	-	-	0	1 (4,57E-07)	-	-	-	0
LA-5	0	-	-	-	-	3 (3,56E-04 ±2,57E-04)	2 (3,52E-04 ±2,43E-04	-	-	-	0

Table S6. Strain-specific qPCR data in placenta, umbilical cord and umbilical cord blood samples, no of subjects with detectable strains and (quantity target per g material)

	Infant seeding group				Maternal seeding group			
	Bifin02	BB-12	LGG	LA-5	Bifin02	BB-12	LGG	LA-5
Placenta	0	0	0	0	0	0	0	0
Umbilical cord	0	0	0	0	0	0	0	0
Umbilical cord blood	0	0	0	0	0	0	0	0

Table S7. Infant growth and tolerability assessments

Safety & tolerability assessments	Infant Seeding (n=23)			Maternal Seeding (n=24)		
	Day 0-14	Day 15-28	Day 29-42	Day 0-14	Day 15-28	Day 29-42
Weight, g (mean±SD)	4032±591	4686±560	5198±554	3944±591	4626±784	5106±784
Length, cm (mean±SD)	55.0±2.1	56.5±2.1	58.8±2.2	54.0±2.0	56.2±2.6	58.0±2.6
Head Circumference, cm	36.4±1.3	37.8±1.2	38.8±1.1	35.8±1.4	37.5±1.4	38.3±1.2
Fever (>38°C), n (%)	1 (4.3)	0 (0)	0 (0)	2 (8.3)	0 (0)	0 (0)
Runny nose, n (%)	2 (8.7)	0 (0)	0 (0)	0 (0)	1 (4.2)	0 (0)
Cough, n (%)	3 (13)	2 (8.7)	2 (8.7)	0 (0)	1 (4.2)	2 (8.3)
Wheezing, n (%)	1 (4.3)	0 (0)	0 (0)	0 (0)	0 (0)	0 (0)
Problems swallowing, n (%)	0 (0)	0 (0)	0 (0)	0 (0)	0 (0)	0 (0)
Rash, n (%)	0 (0)	0 (0)	0 (0)	0 (0)	0 (0)	0 (0)
Problems eating, n (%)	1 (4.3)	0 (0)	0 (0)	1 (4.2)	0 (0)	0 (0)
Retching, n (%)	0 (0)	0 (0)	0 (0)	0 (0)	0 (0)	0 (0)
Vomitting, n (%)						
Bloody vomit, n (%)	0 (0)	0 (0)	0 (0)	0 (0)	0 (0)	0 (0)
Spitting up, n (%)	4 (17.4)	6 (26.1)	7 (30.4)	0 (0)	4 (16.7)	1 (4.2)
Antibiotic use, n (%)	2 (8.7)	0 (0)	0 (0)	2 (8.3)	0 (0)	1 (4.2)
Ilness, n (%)	2 (8.7)	1 (4.3)	2 (8.7)	5 (20.8)	0 (0)	1 (4.2)
